# Second Tier Molecular Genetic Testing in Newborn Screening for Pompe Disease: Landscape and Challenges

**DOI:** 10.3390/ijns6020032

**Published:** 2020-04-05

**Authors:** Laurie D. Smith, Matthew N. Bainbridge, Richard B. Parad, Arindam Bhattacharjee

**Affiliations:** 1 Department of Pediatrics, UNC Hospitals, Chapel Hill, NC 27599, USA; laurie_d_smith@yahoo.com; 2 Laboratory Services Division, Baebies, Inc., Durham, NC 27709, USA; 3 Codified Genomics, Houston, TX 77004, USA; mbainbridge@rchsd.org; 4 Rady Children’s Institute for Genomic Medicine, San Diego, CA 92123, USA; 5 Department of Pediatric Newborn Medicine, Brigham & Women’s Hospital, Harvard Medical School, Boston, MA 02115, USA

**Keywords:** newborn screening, lysosomal storage diseases, variant cut-off, next generation sequencing, diagnosis, dried blood spots

## Abstract

Pompe disease (PD) is screened by a two tier newborn screening (NBS) algorithm, the first tier of which is an enzymatic assay performed on newborn dried blood spots (DBS). As first tier enzymatic screening tests have false positive results, an immediate second tier test on the same sample is critical in resolving newborn health status. Two methodologies have been proposed for second tier testing: (a) measurement of enzymatic activities such as of Creatine/Creatinine over alpha-glucosidase ratio, and (b) DNA sequencing (a molecular genetics approach), such as targeted next generation sequencing. (tNGS). In this review, we discuss the tNGS approach, as well as the challenges in providing second tier screening and follow-up care. While tNGS can predict genotype-phenotype effects when known, these advantages may be diminished when the variants are novel, of unknown significance or not discoverable by current test methodologies. Due to the fact that criticisms of screening algorithms that utilize tNGS are based on perceived complexities, including variant detection and interpretation, we clarify the actual limitations and present the rationale that supports optimizing a molecular genetic testing approach with tNGS. Second tier tNGS can benefit clinical decision-making through the use of the initial NBS DBS punch and rapid turn-around time methodology for tNGS, that includes copy number variant analysis, variant effect prediction, and variant ‘cut-off’ tools for the reduction of false positive results. The availability of DNA sequence data will contribute to the improved understanding of genotype-phenotype associations and application of treatment. The ultimate goal of second tier testing should enable the earliest possible diagnosis for the earliest initiation of the most effective clinical interventions in infants with PD.

## 1. Introduction

Newborn screening (NBS) for Pompe disease (PD), utilizes dried blood spots (DBS) to detect deficient alpha-glucosidase (GAA) activity. As part of currently employed NBS algorithms for PD screening in public health laboratories (PHLs), a first tier biochemical test based on MS/MS or digital microfluidics measures the enzyme activity. The disease is suspected if the enzyme activity is below a previously established cut-off value [1]. However, without two tiered or reflex testing, programs using only first tier biochemical assays would yield poor positive predictive values with high false positive rates or risk high false negative rates due to stringent first tier cut-offs [2]. False-positive results on first tier testing of PD create a high referral burden, often related to the prevalence in the population of non-disease causing pseudodeficiency alleles that lead to low enzyme activity without causing PD. Inclusion of a second tier test, on first tier positive NBS samples provides an opportunity to resolve these false-positives prior to reporting, thus avoiding re-contact, needless referrals and creation of parental anxiety. When GAA activity is below established cutoff values, a second tier test can confirm or disprove the diagnosis of PD. Current algorithms applied by NBS programs may perform repeat sampling and redo the GAA enzyme activity test [3]; or use a second tier test (in-house or as a send-out service) such as DNA sequencing that uses traditional DNA Sanger sequencing technique [4], or the newer targeted next generation sequencing (tNGS) method. Recently, a new second tier test marker—the ratio of creatine/creatinine to alpha-glucosidase activity—has also been proposed [5]. 

From a clinical standpoint, PD has a wide spectrum of phenotypes ranging from early onset with muscle and cardiac involvement (infantile-onset PD; IOPD) to later juvenile or adult onset (later-onset PD; LOPD). LOPD symptomatology may overlap with other neuromuscular disorders, and timely diagnosis is challenging for both forms of the disease. Most early onset cases are symptomatic in some form at birth. Two NBS algorithms for diagnostic confirmation have been developed by a group of international experts on both NBS and PD, the Pompe Disease Newborn Screening Working Group, based on whether DNA sequencing is performed as part of the screening algorithm [6]. Applying the recommendations of either algorithm can lead to a diagnostic characterization as: (a) classic IOPD, (b) “predicted” LOPD, or (c) no disease/not affected/carrier. In both algorithms, a variety of clinical tests are necessary to confirm the diagnosis and generate a treatment plan, including DNA sequencing, since the *GAA* gene variant analysis is essential for confirming the diagnosis and developing a treatment strategy of PD [6]. A challenge to pursuing DNA sequencing as part of NBS algorithms is that many NBS PHLs and clinical referral centers do not perform or have ready access to sequencing resources on premises. When an NBS laboratory does not provide rapid DNA sequencing results through either send-out services or sequencing on premises, the responsibility of obtaining the *GAA* gene sequencing result that is necessary for diagnosis and treatment initiation falls directly on the referral center. Delays in obtaining DNA sequencing results may lead to poorer outcomes or possible loss to follow-up. Loss to follow-up is a concern given the spectrum of phenotypes, many of which present with a delay in onset of symptoms and loss in benefit opportunity of early treatment. If an infant is found to have any form of disease, follow-up with appropriate treatment is necessary and should start early. Recent reports on IOPD continue to shed light on some of the unique challenges care providers face in diagnosing and managing this genetic disease [2,7]. While delays in PD symptom onset and diagnosis are less common in IOPD (under 3–4 months) compared to LOPD, based on the Pompe Registry data [8], the IOPD cases with cross-reactive immunological material (CRIM), negative statuses almost invariably develop high antibody titers to enzyme replacement therapy (ERT). As high antibody titers to ERT reduce the effectiveness of ERT treatment, CRIM-negative patients require immunomodulation therapy prior to initiation of ERT. If CRIM negative IOPDs are not identified in the newborn period, and if immune modulation therapy and ERT treatment are not initiated within days of birth, 100% ventilator free-survival is unlikely, and death may occur within the first two years of life [2,9]. Several Australian IOPD cases that were not identified by NBS, but identified by clinical examination alone have responded poorly to ERT and developed high antibody titres [7]. CRIM-positive IOPDs can also show high antibody titres, which may be predicted based on genotype [10,11]. Rapid recognition of PD at the molecular genetics level, in conjunction with clinical characterization, can guide treatment strategies that may include ERT for IOPD (with or without immune modulation therapy) and LOPD, and may be critical to ensuring the best patient outcomes while preventing irreversible clinical changes [2,12,13]. Given the clinical workup necessary after NBS to confirm a PD diagnosis and direct therapeutic strategy, and the time sensitive nature of the treatment in some PD cases, it may be imperative to also choose a second tier tNGS test that aligns with clinical algorithms and avoids delayed initiation of beneficial PD treatment. Stepwise serial testing between NBS and referral centers, can lead to obtaining DNA results in weeks, delaying initiation of treatment. The typical confirmatory diagnostic DNA sequencing test takes 3–6 weeks and may require reimbursement authorization, and therefore, is inadequate for the prompt genetic testing required for PD. If rapid DNA sequencing testing is included as part of NBS second tier testing, such delays are avoided. Furthermore, such testing may be more equitable for individuals who have no insurance coverage for post-NBS DNA sequencing test. Such decisions, of course, have cost implications for NBS programs.

We therefore also present the rationale for optimizing second tier molecular genetic testing emphasizing (a) fast turn-around time; (b) inclusion of variant effects such as cross-reactive immunological material (CRIM) status, predicted age of symptom onset and heuristics that impact treatment strategies; (c) incorporation of copy number variation and library preparation methods from DBS as part of a tNGS second tier algorithm; and (d) variant prioritization ‘cut-off’ tools to reduce the number of false positives and to help with PD phenotype prediction. Challenges in addressing health information privacy, policy, regulation, parental consent, and secondary or incidental findings associated with genetic tests are topics beyond the scope of this review. 

## 2. Current Approach to Second Tier and Follow-Up Testing

The Taiwanese PD NBS algorithm measures GAA, neutral α-glucosidase (NAG), and maltase-glucoamylase (MGA) activities in separate fluorometric assays, using commercially available fluorogenic (4-methylumbelliferone) substrates [2] normalized to protein concentrations. These values discriminate between true GAA deficiency-confirmed positives from false-positives. The percentage of acarbose inhibition as a second tier test, for samples with inconclusive results, further improves the performance of testing in this algorithm. 

A second tier test marker for NBS of PD recently reported by Tortorelli et al. [5] describes a reduced false-positive rate through the use of a ratio calculated between the creatine/creatinine (Cre/Crn) ratio as the numerator and the activity of acid α-glucosidase (GAA) as the denominator. This ratio is incorporated alongside a post-analytical tool: Collaborative Laboratory Integrated Reports (CLIR; https://clir.mayo.edu) and routinely used in second tier evaluations to address the issue of false positives due to pseudodeficiency. 

Because DBS GAA enzyme activity measured in the first tier NBS cannot predict PD phenotype, additional follow-up evaluation using chest X-ray, electrocardiogram (EKG), creatine kinase (CK) levels, pro-B-type natriuretic peptide (pro-BNP) levels, echocardiogram and genotyping or DNA sequencing (to confirm the presence of two pathogenic variants) is required. Western blot analysis of cultured skin fibroblast lysates has been the gold standard for determining cross-reactive immunologic material (CRIM) status [14], although rapid blood-based assays are also available. Other follow-up tests such as urinary Glc4 levels are also recommended [2,6].

## 3. The Variant Spectrum of Pompe Disease

PD is an autosomal recessive disorder resulting from two pathogenic variants, including multi-exonic deletions, in the GAA gene. The variant spectrum in the *GAA* gene is highly heterogeneous. To date, over 500 pathogenic (P) or likely pathogenic (LP) variants and numerous benign variants and variants of unknown significance (VUS) in the *GAA* gene have been reported (ClinVar, 2017; Erasmus MC University Medical Center, 2017; Aggregation Databases (ExAC, gnomAD), 2017; Leiden Open Variation Database (LOVD), 2018; The Human Genome Mutation Database (HGMD), 2017). A review of *GAA* variants suggests that missense variants are the most frequent molecular genetic cause of PD (~50%), followed by small deletions [8,15]. Variant hotspots are also known, such as the c.-32-13T>G splice site single nucleotide variant and the exon 18 deletion (c.2481+102_2646+31del). These and other ethnic variants are well described [8,15], but require detection by DNA sequencing methods that can identify both single nucleotide variations (SNVs), as well as copy number variations (CNVs, also known as deletion/duplication events), and these are discussed later. 

## 4. Hybrid Capture tNGS as a Second Tier Method

**Figure 1:**
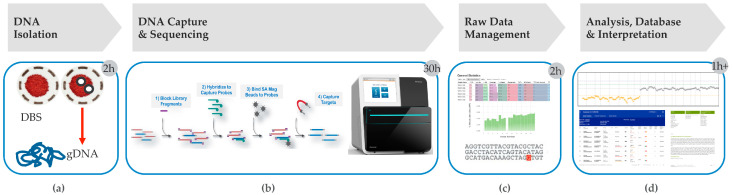
Hybrid Capture-Sequencing tNGS Workflow. Many components of the workflow may be automated: (**a**) DNA Isolation—This step in the workflow may be performed with two 3.2 mm DBS punches, takes 2 h (h) to perform and is an essential requirement of second tier testing as most tNGS protocols call for 2–10 mL of blood; (**b**) DNA hybrid Capture and Sequencing—DNA capture requires library preparation and bait based DNA hybridization-based capture of target regions, which is followed by DNA sequencing (Illumina MiniSeq). (**c**) Raw data management—Involves moving data across secure compliant environments for storage, processing and linking into databases; (**d**) Analysis, database and interpretation—Involves analysis for single nucleotide variants and copy number variant callers, comparative analysis with other database annotations, interpretation and reporting. This overall process takes ~35 h. Adapted from Bhattacharjee et al. [16].

For NBS programs utilizing second tier DNA sequencing, both tNGS on small panels of genes and exome sequencing are considered (see Section 9). Cost and time efficiency needs, along with specimen requirements and future expansion needs, make DNA hybrid-capture based sequencing protocols for tNGS ideal for PD screening/diagnostic algorithms (see Section 5 below for additional considerations). A reduced time through the pipeline due to a more efficient process means shorter time to results and needed treatment for affected infants. A hybrid-capture based tNGS workflow [16], as outlined in Figure 1, may be performed on 1–2 newborn DBS punches, with end-to-end testing time from sample receipt to initiating reports of ~35 h (Figure 1a). In our experience, this approach used for the NBS second tier testing of PD and other lysosomal storage disorders leads to the return of results in less than a week. Reagents used are typically ‘kit-based’ and the processing protocol may be optimized specifically as needed. Most send-out DNA sequencing services typically have a turn-around time of several weeks, as well as referral and sample redraw, making this process challenging for the rapidity needed in NBS protocols. In our experience of processing second tier tNGS for several US PHLs, we have been able to report variant information in seven days, and reduce the referral burden in some cases by 70% (reports that did not have any P/LP variant or had exclusively pseudodeficiency variants). These reports, therefore, were capable of providing carrier status, CRIM prediction, and severity of the known reportable variants, which would not otherwise be possible.

**Figure 2:**
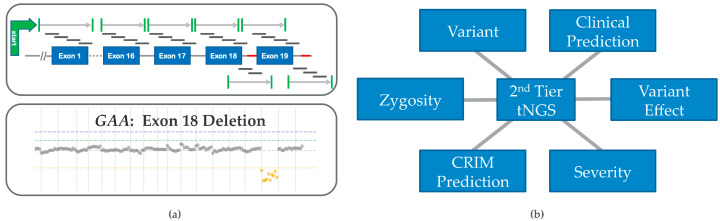
Second tier Testing features: (**a**) Top panel shows how *GAA* gene structure is targeted for sequencing with overlapping single stranded DNA or RNA probes of 60–200 nucleotides in target exonic regions (blue rectangles), adjoining splice junctions (marked in green), and known hotspots (red). The bottom panel shows the results of a in-house algorithm that integrates detection of copy number variation (CNV) or del/dup events in Pompe disease, such as the *GAA* exon 18 deletion; (**b**) 2nd tier tNGS provides variant (genotype) information for better clinical predictions and management of care. Pseudodeficient alleles, variant effect, and cross-reactive immunologic material (CRIM) prediction can be elucidated from previously observed data [8,14].

DNA library preparation involves sonication or a kit-based enzymatic DNA fragmentation step (amenable to automation and high throughput) followed by subsequent adaptor ligation/indexing. Indexed DNA fragments are then hybridized to custom DNA enrichment probes in order to target the specific genes on the panel (Figure 1b). Such DNA probes are available from various commercial sources and may be custom designed to target 100–200 genes. Sequencing may be performed on several sequencing platforms. The Illumina MiniSeq platform shown in the example (Figure 1b), takes around 14h to complete a run and can parallel process 20 samples per run. The bioinformatics workflow (raw data management) is processed mostly in parallel and can take an additional 2 h. Custom QC metrics provide insight into sequence quality for each sample as well as coverage gap analysis (Figure 1c). A custom copy number variation (CNV) caller can be used on the same sequence dataset to identify CNVs (Figure 1d, Figure 2a). Strict quality metrics prevent CNV calling on samples with low coverage. An in-house variant database with a well-established analysis platform, such as from Opal^TM^ (Fabric Genomics), can appropriately annotate the data set in real-time and help generate sequencing reports more efficiently. Automated variant scoring allows a reduction in time for data interpretation, and thus drives higher throughput. 

## 5. Ideal PD Second Tier tNGS Testing Features

### 5.1. Timeliness

The US Federal and State Public Health programs emphasize the importance of timeliness of NBS, setting benchmarks for the efficient collection, transportation, testing, and reporting of results. First and second tier tests are combined to optimize detection of cases at a false positive rate as low as possible and keep overall costs of the combined tests low. Upon referral, necessary diagnostic tests are done. For many diseases that progress slowly, the split in the burden between screening and diagnostic phases may be sufficient. However, this tiered/serial testing and patient recall can cause delays in time-sensitive PD. For most of the LOPD cases that progress slowly, this approach would be sufficient without causing harm. However, for suspected IOPD cases, time to treatment initiation within two weeks is critical. Therefore, timely second tier tNGS reporting in IOPD may be the only option for avoiding diagnostic and treatment delays and avoiding a negative impact on quality of life and potentially on lifespan [7,13]. The treating clinician, in the case of classic IOPD, would need DNA sequencing data in the diagnostic-phase to determine treatment-associated predictive sequence information (CRIM status and phenotype onset predictions) prior to initiating enzyme replacement therapy [2,6]. Although controversial, suspected LOPD patients identified by NBS second tier tNGS may also benefit from follow-up and earlier intervention [2,13,17]. Rapid return of results is a frequent claim by providers of DNA sequencing for diagnostic testing. However, the DNA sequencing turnaround time is generally inadequate for early onset PD (minimum industry standard is three weeks’ turnaround). Such inefficiencies make utilization of most commercial resources untenable for the required timeframe of treatment following the diagnostic phase. Second tier tNGS workflow services, if performed end-to-end, may be completed in ~35 h, and reports can be finalized consistently within 5–7 days of receiving the sample. If NBS programs routinely perform tNGS on premises, or use DNA sequencing services that return results within 7 days, then overall reporting and treatment initiation may occur within 10 days, benefiting classic IOPD or CRIM-negative IOPD cases. Starting ERT within this timeframe for CRIM-negative IOPD, and before the immune system has matured, might also enable natural immune tolerance [7]. Omitting the tNGS assay as the second tier PD NBS test and putting the burden on the follow-up referral centers to obtain a molecular diagnostic result, while providing a faster NBS turn-around time may delay treatment initiation. Due to the fact that the optimal effectiveness of ERT in IOPD hinges on the prompt identification of two *GAA* pathogenic variants in-trans [6], a second tier tNGS assay may actually be beneficial by shortening the time to treatment initiation. Identification of one or two pathogenic variants (without *cis* or *trans* determination), combined with a low GAA activity is sufficient to consider need for further confirmatory testing. 

### 5.2. Prediction of Genotype-Phenotype Effects

These topics have been well covered by other reviews [8,15], and therefore only discussed in the context of second tier testing algorithm for PD NBS (Figure 2). In PD, the genotype is generally fully penetrant; IOPD phenotypes especially demonstrate limited heterogeneity. A pseudodeficiency allele is always a pseudodeficiency allele as it causes low measured GAA activity that does not change from individual to individual. However, predicted CRIM status may not exactly correlate as observed in some patients with Western blot analysis of cultured skin fibroblast lysates or for splice variants and may require additional studies [14]. Identification of variants in the *GAA* gene provides valuable information for determining variant-phenotypes, establishing genotype–phenotype correlations, confirming diagnosis or carrier status and counseling about the significance of these findings [6]. Measuring first tier GAA activity, genotyping, and determination of CRIM status from genotype (important for phenotype onset prediction) is necessary to start therapy [14,18]. 

### 5.3. Prediction of CRIM Status

In general, for babies with IOPD who have cardiac involvement, treatment with recombinant human GAA (rhGAA) is initiated immediately after confirmation of GAA deficiency and positive CRIM status, either by genotyping prediction or by western blot [2,6]. ERT is started after the cardiac involvement is confirmed, unless immunomodulation therapy to prevent anti-GAA antibodies production is planned. For infants without cardiac involvement, close follow up is needed and ERT treatment is delayed until symptoms appear. As confirmatory biochemical tests and assays are not readily available for PD, and additional testing may delay initiation of therapy, sequence variant identification may be useful to predict CRIM status, thereby directing treatment decisions [14,18]. The presence of two nonsense or frameshift-termination variants is a good predictor of CRIM status, typically resulting in CRIM-negative status, unless the premature stop codon is in the last exon of the gene [14]. 

### 5.4. Copy Number Variation (CNV) and False Negative Risk

Due to the fact that CNV events such as those reported in *GAA* exon 18 [8,15] and other heterogeneous CNVs comprise a significant fraction of pathogenic PD variants, a PD NBS second tier tNGS assay must integrate detection of CNV events. While data from targeted small gene panels are scarce, recent reports of PD whole exome sequences provide insight into CNVs. Mori et al. [19] evaluated whole exome sequencing to identify infantile- and late-onset PD (*n* = 93), concluding that some pathogenic variants may have been missed, since the variant pipeline used did not identify variant hotspots such as the exon 18 deletion (Figure 2a), and c.-32–13 single nucleotide variant. A sensitivity of near 100% can be achieved if issues with custom design, gap filling, improving >20X sequence coverage and deletion duplication coverage are addressed.

### 5.5. False Positives Resolved by Second Tier tNGS Testing

The variant burden and its effect on an individual is unknown at birth, and phenotype characterized by biochemical activity alone may be imprecise. Low GAA enzyme activity detected by first tier assays may identify pseudodeficiency cases and carriers of a single pathogenic *GAA* variant as a false positive, thus enzyme activity results alone are not definitive and cannot differentiate between true PD and newborns who carry pseudodeficiency alleles or sometimes PD carriers (P/LP variants). Such cases can be distinguished by genetic testing. 

### 5.6. Other Considerations

Occasionally it may be difficult to resolve a false positive with second tier tNGS. Hypothetically, if a sample has a low enzyme activity value on first tier testing and there are no findings on second tier tNGS, the etiology of the low enzyme activity has not been identified and the result can be called negative in screening, or an additional biochemical test can be done. The etiology of discordant results may involve sample mix up or other technical issues. A sample mix up can sometimes be addressed by comparing imputed sex from tNGS data to that reported on accession data. Monitoring of additional DBS enzyme activity values may also indicate false positive risk. For example, if some additional enzyme activity values are all low, it may suggest issues with stability, storage or transportation. As mentioned, new post-analytical tools, such as Collaborative Laboratory Integrated Reports (CLIR; https://clir.mayo.edu) and second tier biochemical testing are becoming available to address these issues [5,17]. A tNGS false negative result when the first tier has low enzyme value is very unlikely given the recessive nature of PD, where the presence of at least two causal variants in trans is expected. We discuss below the post-analysis of tNGS data, including statistical tools to predict and reduce the number of false positives and understand PD phenotypes. 

## 6. Genome Scale Data and Its Impact on PD Screening

We evaluated how information in public genomic databases would inform and impact PD NBS that utilized second tier tNGS. In order to identify potential pathogenic variants for the detection of PD through NBS, we reviewed *GAA* allele frequencies in the gnomAD database. Based on gnomAD and ClinVar, pathogenic and likely pathogenic (P/LP) variants have a total allele frequency of 0.635%. Assuming these alleles segregate independently and are fully penetrant, the probability of inheriting two such alleles is the square of 0.635 or 0.004%. This roughly equates to an expected incidence of 1/24,780 births, which is in the expected range for PD (1/14,000 to 1/100,000). This means that a significant proportion of the variant alleles that cause PD are known and can be automatically identified in a high-throughput fashion.

For the sake of timeliness, it is critical to have a tNGS assay that can identify all genetic variations in a single test. To assemble relevant variants for the diagnosis of PD through NBS, we reviewed alleles in the gnomAD database for potential CRIM status. GnomAD’s *GAA* sequences from 141,456 individuals revealed 213 (0.15%) predicted LOF variants (pLOF) at 82 unique sites, of which approximately 50% were frameshift termination and 25% each were splice-site and stop-gain variants. None of these mutations were present in a homozygous state and although an additional mutation may be present in trans; it is more likely that these individuals are presumed carriers. Based on gnomAD data, the carrier frequency of unique pLOF variants is 0.15% (213/141,456) in the population, which is 4-fold less than P/LP variants in gnomAD. Estimates of carriers in general uncovered by NBS is a fraction (0.007–0.013%) of that observed frequency in gnomAD [17]. Although some may be, most PD carriers will not be detected by NBS program based on pLoF or P/LP variant frequencies in gnomAD. While reporting on carrier status is not the aim of most NBS programs (most try to avoid detecting carriers), parents of affected PD patients are obligate carriers. The risk for two carrier parents to have an affected child is 25% with each pregnancy. Thus, the mode and impact of reporting carrier status to parents after detection through a PD tNGS second tier NBS algorithm require careful consideration. Other NBS disorders deal with this issue routinely (e.g., cystic fibrosis carriers via a DNA based second tier, or sickle cell anemia via electrophoresis).

A family history of negative PD screens without carrier information gives families a false sense of security, as reported in the Australian PD cases [7]. A significant fraction of FPs will be due to recurrent carriers or pseudodeficiencies with low enzyme values, and a likely source of anxiety or rightful concern in future newborn births in those families. Thus, a delay in relaying carrier or pseudodeficiency information may have secondary consequences that may need to be considered. Recently, the BabySeq Project (a part of NSIGHT), evaluated newborn diseases using genome scale sequencing in a randomized clinical trial pilot. The cohort studied included both well newborns and those admitted to a neonatal intensive care unit [20,21]. In a cohort of 159 newborns that underwent genome scale NGS based whole exome sequencing (WES), one carrier of a pathogenic *GAA* variant was identified. As part of that study protocol, the in-person genetic counseling was provided to the family. 

We also queried 14,821 WES individuals referred for clinical genetic testing to determine a ‘hit rate’ for *GAA*, generating a list of reportable variants by curating pathogenic and likely pathogenic variants from reputable sources in ClinVar and from manual literature curation. These estimates are useful in understanding PD causal variant frequencies and database quality. This curation resulted in over 100 *GAA* reportable variants. For each case, the exome variant file (variant call format (VCF) file) was scanned through our database and scored as to whether that sample was identified (“hit”). Only one homozygous hit (allele fraction > 90%) was observed for *GAA*. Due to the fact that testing was performed on single samples, it was not possible to know if multiple variants were in cis or trans. Thus, we defined a compound heterozygous hit as having two or more variants in a single gene, in a single sample, from our reportable list of variants. Only two cases had reportable variants in *GAA* and thus the potential “hits” for the referred population was 2/14,821 (0.014%), a value that was consistent with the incidence of PD. These types of analyses have several limitations: (a) no ethnicity control was available, as ethnicity of patients was not known, and therefore impact on actual screening could be variable; (b) consanguinity of patients was not known; (c) patients in the dataset were likely depleted of traditional PD; (d) it was not possible to distinguish the cis and trans configuration of multiple hits in a single individual; (e) this analysis did not take into account novel variants, nor did it take into account copy number variants; (f), variant calls were affected by coverage of the gene/base and particularities of the caller. Thus, while it was not possible to perfectly match the false positive /false negative rate of the caller, this effect was likely small.

## 7. Variant ‘Cut off’ for PD

To reduce the burden of interpreting variants of uncertain significance (VUS), we considered a ‘cut off’ model to filter variants that are too common in population to cause disease. We estimated the maximum credible allele frequency (MCAF), using a statistical framework that was previously reported for a recessive inheritance model by Wiffin et al. [22] Variants that occur more frequently than the MCAF cutoff should not be considered as a causative variant for the disease. 

The equation to calculate MCAF is as follows (1):

MCAF = sqrt(*v*) × *c* × sqrt(*g*) × 1/sqrt(*p*) (1)

where *c* is the maximum allelic contribution, which represents the proportion of cases that are attributable to the gene that is attributable to an individual variant, *g* is the maximum genetic contribution representing the proportion of all cases that are due to the gene, *v* is the prevalence and *p* is the penetrance. This value can be further refined by assuming that the most common causative alleles are known. 

For the *GAA* gene, we identified common alleles that were both expertly curated and present in ClinVar. Furthermore, we established a set of trusted ClinVar submissions (multiple sites with no conflicts (GeneDx and Invitae)) and identified all P/LP variants. From this we identified the most common pathogenic variants that contribute to the disease (ClinVar and trusted submitters). We then made the assumption that once the most common variants are accounted for, the maximum allelic contribution of any additional variant is 5%. We use 0.000025 (1/40,000) as the incidence of high-risk PD, 1 as the penetrance and assume all causes of PD are attributable to variants in *GAA*. The calculation for MCAF is then: sqrt(0.000025) × 0.05 × sqrt(1) × 1/sqrt(1) or 0.00025. We then compare this to the maximum MAF in manually curated or trusted submitter (Table 1). This value can then be used to remove likely spurious entries in ClinVar from further consideration. Furthermore, novel variants (VUSes) with MAFs above this cut-off can likely be discarded as likely benign.

## 8. Follow-Up Infrastructure for Families, Screen Positive Infants and Carrier Status 

Some of the most difficult issues generated by NBS for PD will be the follow-up care, for not only the infants who screen positive, but also for the parents and families. As of 2017, the Pompe Disease Newborn Screening Working Group has proposed options for including DNA sequencing as part of the screening algorithm or follow-up [6]. The Pompe Registry, a long-term, multinational observational program (NCT002314000), designed to improve understanding of the natural history and outcomes of patients with PD, started in 2004, and is sponsored and administered by Sanofi Genzyme (Cambridge, MA), the pharmaceutical company that markets PD ERT. The Pompe Registry has *GAA* variants and phenotypes for 1079 patients [8]. Despite the existence of this network, challenges surround the referral of those patients to appropriate care centers, to ensure that up to date care guidelines that reflect the current standards of the PD NBS community are adhered to. Given that current disease modifying treatments for PD may depend on knowing the specific genetic defect, it is critical and ethically required, that all screen positive infants have timely access to genetic testing, as either part of NBS or as referral within the timeframe. Testing should define the specific variants and access to appropriate follow up clinical expertise, so that the appropriate treatment is provided. Future therapies, once approved, such as chaperones, c.-32-splice switching antisense oligonucleotides and AAV-directed gene therapies will need variant information.

Testing parents to determine which parent carries which PD variant is necessary to provide appropriate counseling regarding future reproductive risk for family members. Knowing familial variants for PD can be useful for reproductive planning and the avoidance of recurrence, but is not the primary goal of NBS. Most guidelines recommend against carrier testing of minors and minor siblings, as there is no medical benefit until reaching reproductive age and age of consent [23,24,25]. 

Some screen positive infants will have an early-onset form of the disease while others will develop LOPD. Knowing the precise genetic defect in a screen positive IOPD case is critical for the prediction of the long-term clinical outcome of ERT and the development of antibodies against the infused enzyme. An analysis of CRIM status for every IOPD patient within the first two weeks of life is essential. For this reason, sequence analysis of the GAA gene may be justified as a tiered testing step for PD NBS programs or a rapid turnaround confirmatory test for the referral center. Some first tier screen positive newborns may have resolution on a tNGS second tier test, as they may carry pseudodeficiency alleles or a variant burden, that are otherwise low risk. For parents of screen positive infants with identified variant(s), familial testing is relatively simple and may be considered in reproductive planning. Normal GAA enzyme activity, or an above threshold value on a PD NBS, does not confirm a non-carrier status. It is possible that a pathogenic variant may have been missed or that a phenotype may manifest later. The potential therefore exists for misconstruing a negative PD NBS. Further studies must evaluate whether all infants identified with disease or as carriers have received adequate follow-up, including diagnostic confirmation after a positive screen, referral to appropriate clinical care centers and delivery of best practice treatment and management across the lifespan. State NBS programs must have a short-term follow-up plan in place to ensure tracking of screen positive newborns for the receipt of proper referral to appropriate care centers for diagnosis and treatment. Ideally, this short-term follow-up program should have access to resources that include: (a) *GAA* sequencing availability or referral for rapid turnaround DNA sequencing for screen positive infants; (b) the ability to provide cascade genetic testing of both family and extended family members; and (c) the ability to carry out all necessary confirmatory testing. Furthermore, referral sites should be able to appropriately triage infants found to have incidental findings like other overlapping neuromuscular diseases, such as Limb Girdle muscular dystrophy. Since it is recommended that CK levels be determined in all infants in whom 1st and 2nd tier testing is suspicious of PD, there will likely be utility in determining baseline CK levels from DBS at the time of initial enzyme testing. This would be helpful not only for confirming the diagnosis and establishing a baseline level prior to starting treatment, but also allowing for early identification of other neuromuscular disorders if there is persistence of hyperCKemia, despite negative testing for PD. As with all genetic testing, identifying and implementing appropriate educational programs is key. Improving health care provider and parental genetic literacy about genetic disorders is required. 

## 9. Current and Future Utilization of tNGS Testing

PD was added to the U.S. Recommended Universal Screening Panel (RUSP) in 2015. Targeted NGS could reduce the treatment delay for those identified by PD screening by allowing analysis, interpretation, and appropriate reporting of healthcare related information in a timely manner. Already several PHLs in the USA have adopted second tier tNGS in-house or as a service [26,27]. New York and California PHLs have strong DNA sequencing second tier programs including tNGS for PD and other NBS disorders. Several other NBS PHLs use ‘send-out second tier tNGS services’ for PD. Given developments in genome scale technologies, such as WES or whole genome sequencing (WGS), the utilization of tNGS may be co-opted for needs in second tier testing of PD. Recently, the Utah PHL has started implementing a second tier exome sequencing protocol for genes associated with newborn screening abnormalities [28]. Furthermore, tNGS based testing can avoid constraints or complicating factors associated with biochemical testing, such as the infant’s gestational age at birth, transfusion status, age at sample collection, need for repeat sampling (rescreens or redraws), and metabolic and feeding states. The NIH funded NSIGHT consortium (Newborn Sequencing in Genomic Medicine and Public Health) evaluated newborn diseases using genome scale sequencing in randomized clinical trials [21,22,29]. This is an exciting time to be at the forefront of applying genomic information to rapidly identifying and treating genetic disorders such as PD. We envision that population level screening as first tier WES or WGS, or even first tier small gene panel based tNGS testing, is unlikely to be the avenue by which PD may be identified in the near term, due to considerations such as cost and complexity. However, with improvements in technology and a decrease in cost, this may likely be the NBS of the future.

## 10. Conclusions

In summary, molecular based NGS testing approaches are suitable for NBS of PD, and may be used in algorithms that include either a tandem second tier test or in a contingent fashion. The primary goal of NBS is identifying patients who can be treated to establish significant health gain. Secondary goals, such as shortening the diagnostic odyssey, identifying carriers and providing information for reproductive options are of lesser concern. However, as PD is a spectrum disease simply providing biochemical screen positive information alone delays treatment initiation in those phenotypes with rapid disease progression, which impacts the ultimate outcome. PD has both pseudodeficiencies as well as early-onset and late-onset clinical phenotypes, presenting a special challenge to families and medical follow-up centers. Early genotype information in PD management allows for prompt treatment initiation and the potential for better clinical outcomes, especially in infantile onset disease [2,3,9,12,13]. Considering the recommended diagnostic algorithm [2,6], of which DNA sequencing is a part, the use of second tier tNGS sequencing is only logical, irrespective of whether the PHL yet has sequencing capabilities, since NBS may be the only way to provide molecular genetic information for early determination and treatment of PD in an equitable manner. Several PHLs including New York and California have already introduced DNA sequencing in their PD NBS algorithms. While second tier biochemial tests are capable of PD identification, second tier tNGS test for PD can provide rapid and precise information on highly penetrant recurrent pathogenic variants, distinguish pseudodeficiency alleles with lower biochemical values that are false-positives, provide clues for CRIM status, and identify variants associated with IOPD or LOPD phenotypes. A combination of early detection, close monitoring, and early ERT is likely to be beneficial to LOPD patients, but additional data are needed. Consistent with the original intent of NBS, PD must be considered a time critical condition, for which all PD NBS results including second tier should be provided as early as possible. Given the ever-increasing population-based variation outcome data for PD [30], implementation of tNGS second tier testing of PD from a DBS [16], is both feasible and sufficient in the required time frame for NBS and follow up.

## Figures and Tables

**Table 1:** Maximum Credible Allele Frequency (MCAF): Allelic Contribution, Penetrance, MAF cutoff, pathogenic variants in ClinVar from Trusted submitters and final MAF cutoff.

Prevalence	Max. Allelic Contribution	Penetrance	MCAF (Whiffin et al. [22])	Max. MAF in ClinVar	Final Cut Off
0.000025	0.05	1	0.00025	0.00358	0.00025
